# Phosphoproteome profiling of mouse liver during normal aging

**DOI:** 10.1186/s12953-022-00194-2

**Published:** 2022-08-05

**Authors:** Jiang-Feng Liu, Yue Wu, Ye-Hong Yang, Song-Feng Wu, Shu Liu, Ping Xu, Jun-Tao Yang

**Affiliations:** 1 State Key Laboratory of Medical Molecular Biology, Department of Biochemistry and Molecular Biology, Institute of Basic Medical Sciences, Chinese Academy of Medical Sciences & Peking Union Medical College, Beijing, 100005 China; 2 School of Statistics and Data Science, Nankai University, Tianjin, 300071 China; 3 State Key Laboratory of ProteomicsResearch Unit of Proteomics & ResearchDevelopment of New Drug of Chinese Academy of Medical Sciences, Institute of Lifeomics, Beijing Proteome Research Center, National Center for Protein Sciences (Beijing), Beijing, 102206 China

**Keywords:** Normal aging, Mouse, Liver, Phosphoproteome, Label free

## Abstract

**Background:**

Aging is a complex biological process accompanied by a time-dependent functional decline that affects most living organisms. Omics studies help to comprehensively understand the mechanism of aging and discover potential intervention methods. Old mice are frequently obese with a fatty liver.

**Methods:**

We applied mass spectrometry-based phosphoproteomics to obtain a global phosphorylation profile of the liver in mice aged 2 or 18 months. MaxQuant was used for quantitative analysis and PCA was used for unsupervised clustering.

**Results:**

Through phosphoproteome analysis, a total of 5,685 phosphosites in 2,335 proteins were filtered for quantitative analysis. PCA analysis of both the phosphoproteome and transcriptome data could distinguish young and old mice. However, from kinase prediction, kinase-substrate interaction analysis, and KEGG functional enrichment analysis done with phosphoproteome data, we observed high phosphorylation of fatty acid biosynthesis, β-oxidation, and potential secretory processes, together with low phosphorylation of the Egfr-Sos1-Araf/Braf-Map2k1-Mapk1 pathway and Ctnnb1 during aging. Proteins with differentially expressed phosphosites seemed more directly related to the aging-associated fatty liver phenotype than the differentially expressed transcripts. The phosphoproteome may reveal distinctive biological functions that are lost in the transcriptome.

**Conclusions:**

In summary, we constructed a phosphorylation-associated network in the mouse liver during normal aging, which may help to discover novel antiaging strategies.

**Supplementary Information:**

The online version contains supplementary material available at 10.1186/s12953-022-00194-2.

## Highlights

The first phosphoproteome profiling of mouse livers during normal aging. A total of 5,685 phosphosites in 2,335 proteins were quantified in this study. A phosphorylation-regulated pathway network was constructed. Metabolism, secretion, and the cell cycle might be dysregulated during normal aging.

**Supplementary Information:**

The online version contains supplementary material available at 10.1186/s12953-022-00194-2.

## Introduction

Aging and aging-associated diseases have imposed great suffering and economic burdens on both individuals and society [1]. Antiaging interventions have been researched for years and the development of modern biology has promoted the discovery of antiaging methods. Hallmarks of aging can be summarized as epigenetic alterations, loss of proteostasis, dysregulated nutrient sensing, cellular senescence, etc. [2]. Several interventions, including senolytics, NAD precursors, metformin, exercise, and caloric restriction, can potentially increase the health span and/or lifespan [3]. However, pivotal antiaging methods are still lacking because our global view of normal aging remains incomplete.

Omics analysis offers the advantage of obtaining an overall profile of biological processes and has been applied in aging-associated research [4]. In this study, we focused on phosphoproteome profiling of mouse liver during normal aging. The liver is the largest metabolic organ in humans and mice. Although the aging liver shows relatively modest physiological changes [5], omics studies have discovered some characteristic molecular alterations, such as remodeled DNA methylation [6], increased inflammation [7, 8], disrupted metabolic homeostasis and circadian metabolism [9]. The phosphoproteome has been used for functional liver research, including circadian control [10], nonalcoholic fatty liver disease and steatohepatitis [11]. Phosphorylation is a quick and flexible way for organisms to respond to physiological changes, and abundant research data have been accumulated. As the phosphoproteome profiling of the liver during aging has not been investigated, we applied mass spectrometry-based phosphoproteomics to obtain the first global in vivo quantification of aging-associated phosphorylation in mouse livers.

## Materials and methods

### Animals

Wild-type C57BL/6 J male mice were allowed access to food and water ad libitum. The colony rooms were maintained at a constant temperature and humidity with a 12:12 light/dark cycle. Two-month-old mice were used in the young group, and eighteen-month-old mice were used in the old group. All animal protocols were approved by the Animal Care and Use Committee of the Institute of Basic Medical Sciences, Chinese Academy of Medical Sciences and Peking Union Medical College.

### Sample preparation

The young and old groups were assigned 15 mice each. During the long feeding period, 4 mice in the old group died naturally. The remaining mice were euthanized by massive bloodletting from the orbital vessel after anesthesia with tribromoethanol. Next, the whole liver was excised from each mouse, immediately dissected, and stored separately. Samples acquired for phosphoproteome and transcriptome analyses were immediately frozen in liquid nitrogen and transferred to -80 ℃ until use. Samples for H&E and Masson analyses were quickly placed in 4% paraformaldehyde (PFA). Samples for oil red O analysis were appropriately embedded into optimal cutting temperature compound (OCT) and stored at -80 ℃ until use. After evaluation, we excluded old mouse with liver tumor and young mouse that looked normal but had light weight because of gastrointestinal flatulence. Thus, 14 samples from young mice and 10 from old mice were finally selected for the follow-up analyses.

### Morphological analysis

#### H&E and Masson staining

Liver samples were fixed in 4% PFA overnight. Fixed tissues were dehydrated by 75% ethanol for 4 h, 85% ethanol for 2 h, 90% ethanol for 2 h, 95% ethanol for 1 h, absolute ethanol for 30 min twice, ethanol-dimethylbenzene for 5 min, and dimethylbenzene twice for 10 min. Dehydrated tissues were embedded in paraffin and cut into 4  m-thick sections. The paraffin-embedded sections were successively placed in dimethylbenzene twice for 20 min each, absolute ethanol for 10 min twice, 95% ethanol for 5 min, 90% ethanol for 5 min, 80% ethanol for 5 min, and 70% ethanol for 5 min, then washed with distilled water.

For H&E staining, hydrated sections were placed in hematoxylin solution for 3–8 min, 1% hydrochloric acid/ethanol differentiation solution for seconds, 0.6% ammonia for seconds, and eosin solution for 1–3 min.

For Masson staining, hydrated sections were processed according to the manufacturer’s protocol of the Masson staining kit (Wuhan Goodbio Technology Co., Ltd, G1006).

The stained sections were subsequently transferred into 95% ethanol for 5 min twice, absolute ethanol for 5 min twice, and dimethylbenzene for 5 min twice. Next, the sections were dried and sealed with neural gum. Pictures were taken with a Nikon Eclipse CI imaging system.

#### Oil red O staining

Liver samples embedded in OCT were moved to a freezing microtome and cut into 8  m-thick sections at -20 ℃. The sections were dried at room temperature for 10 min, fixed with 4% paraformaldehyde (PFA) for 15 min, and washed with phosphate-buffered saline (PBS) for 5 min three times. Sections were transferred into oil red O solution (G1016, Goodbio Technology Co., Ltd) for 10 min, followed by 75% ethanol for 2 s, and then washed with water for 1 min. Next, the sections were transferred to hematoxylin solution for 1 min, 1% hydrochloric acid/ethanol differentiation solution for 3 s, and 0.6% ammonia for 3 s, after which they were washed with water. Excess water was removed, and glycerin gelatin was used to seal the sections. Pictures were taken using a Nikon Eclipse CI imaging system (Japan).

### Phosphoproteome and analysis

#### Protein extraction and digestion

Mouse liver tissue samples were ground in liquid nitrogen and sonicated with lysis buffer (9 M urea, 10 mM Tris–HCl (pH 8.0), 30 mM NaCl, 50 mM IAA, 5 mM Na_4_P_2_O_7_, 100 mM Na_2_HPO_4_ (pH 8.0), 1 mM NaF, 1 mM Na_3_VO_4_, 1 mM sodium glycerophosphate, 1% phosphatase inhibitor cocktail 2 (Sigma, St. Louis, USA), 1% phosphatase inhibitor cocktail 3 (Sigma, St. Louis, USA), and 1 tablet of EDTA-free protease inhibitor cocktail (Roche, Basel, Switzerland) for every 10 mL of lysis buffer). Then the total lysate was centrifuged at 17,000 × *g* for 8 min at 4 ℃ to remove debris. The evaluation of protein concentration and subsequent in-gel digestion were performed as previously described [12]. In brief, 1 mg of proteins was used as starting material for each sample, and the proteins were reduced with 5 mM DTT for 30 min at 45 °C and were alkylated with 20 mM iodoacetamide for 30 min at room temperature in the dark. Next, proteins were resolved on a 10% SDS–PAGE gel, run for a 0.8 cm length, and then stained with Coomassie Blue G-250. The entire gel lane was sliced into 1 mm^3^ pieces and destained, followed by in-gel digestion with 10 ng/μL trypsin (Promega, Madison, WI, USA) at 37 °C overnight.

#### Phosphopeptide enrichment

Phosphopeptides were enriched by the Ti^4+^-IMAC method [13] as previously reported with minor modifications. Briefly, peptide mixtures were resuspended in 500 μL of loading buffer (5% ACN, 50 mM NH_4_HCO_3_) and acidified with 500 μL of binding buffer (80% ACN, 6% TFA), followed by the addition of 30 mg Ti^4+^-IMAC beads. Then, the mixtures were mixed thoroughly on a Vortex Genie2 for 30 min and centrifuged at 17,000 × *g* for 6 min to remove the supernatant. The beads were washed with 1.8 mL of wash buffer 1 (80% ACN, 6% TFA) once and 1.8 mL of wash buffer 2 (80% ACN, 0.1 TFA) twice and all the wash buffers in each step were removed by centrifugation at 17,000 × *g* for 6 min. Afterward, the beads were resuspended in 1 mL of elution buffer (10% NH_3_·H_2_O), and the mixture was vortexed for 15 min and sonicated in ice water for 15 min followed by centrifugation at 17,000 × *g* for 6 min. The eluted supernatant was collected. The beads were vortexed for 5 min in another 500 μL of elution buffer and centrifuged to collect the supernatant. The eluted supernatants were combined and centrifuged at 21,000 × *g* for 8 min to further remove the beads. Then, the supernatant was vacuum dried, and the eluted phosphopeptides were stored at -80 ℃.

#### Stage-tip separation

The homemade C18 Stage-Tip [14] was used to separate the phosphopeptides into 3 fractions. The Stage-Tip was first activated with 40 μL of methanol and then washed twice with 40 μL of wash buffer (80% ACN in 10% NH_3_·H_2_O) and twice with 40 μL of loading buffer (10% NH_3_·H_2_O). The phosphopeptides were resuspended in 40 μL of loading buffer and loaded onto the Stage-Tip. Then, the phosphopeptides were eluted in a sequential gradient of ACN (0% ACN, 2% ACN, 5% ACN, 8% ACN, 10% ACN, 20% ACN, 40% ACN, 50% ACN, 80% ACN) in 10% NH_3_·H_2_O buffer (20 μL/fraction). All 9 fractions were combined into 3 fractions (0% ACN, 8% ACN, and 40% ACN in fraction 1; 2% ACN, 10% ACN, and 50% ACN in fraction 2; 5% ACN, 20% ACN, and 80% ACN in fraction 3). All 3 fractions were lyophilized immediately.

#### LC–MS/MS analysis and database search

The fractionated enriched peptides were eluted on a Thermo Fisher EASY-nLC 1200 liquid chromatograph and analyzed by Orbitrap Fusion Lumos Tribrid MS (Thermo Fisher Scientific). Peptides were separated on a 16 cm column with a 75 μm inner diameter packed with Dr. Maisch GmbH reversed-phase material Reprosil-Pur 120 C18-AQ, 1.9 μm resin (SinoAmerican Proteomics, LLC). Separation was performed using 75 min runs at a flow rate of 300 nL/min with a nonlinear gradient. The elution gradient was as follows: 3–10% B for 3 min, 10–24% B for 55 min, 24–32% B for 10 min, 32–90% B for 4 min, and 90% B for 3 min (Phase A: 0.1% FA and 2% ACN in ddH_2_O; Phase B: 0.1% FA in 80% ACN). The initial MS spectrum (MS1) was analyzed over a range of m/z 350–1500 with a resolution of 60,000 at m/z 400. The automatic gain control (AGC) was set to 4 × 10^5^. The subsequent MS spectrum (MS2) was analyzed using data-dependent mode searching for the top 40 intense ions fragmented in the linear ion trap via high-energy collision dissociation (HCD) with the NCE set to 30. The MS2 resolution was 15,000 at m/z 400. The cycle time was 4 s.

MS/MS raw files were processed in MaxQuant (version 1.5.3.8) against the UniProt *Mus musculus* database (downloaded 2018.04.10, containing 16,972 sequences) for peptide identification, label-free quantification, and phosphosite localization. The parameters set for database searching were as follows: Cysteine carbamidomethyl was specified as a fixed modification. Oxidation of methionine, phospho (STY) and protein N-acetylation were set as variable modifications. The tolerances of precursor and fragment ions were set to 20 ppm. For digestion, trypsin was set as the protease with the utmost two missed cleavages permitted. The false discovery rate was set to 1% at the protein, peptide and modification levels. Matched between runs and label-free quantification were selected.

#### Data management

Proteins/peptides in the reverse decoy database and potential contaminant database were excluded. The localization probability of phosphorylation ranged from 0 to 1, and peptides with localization probabilities of > 0.75 were grouped into Class I and selected for further analysis. Normalization was performed with Perseus (v.1.6.5.0) [15] by dividing the intensity by the median of each group. Using a two-sample *t* test, a site with a p value < 0.05 was considered to be differentially expressed. Up- and downregulated sites were defined by fold-change (FC, mean values of old mice/mean values of young mice) > 1 or < 1, respectively.

#### Functional enrichment analysis

Kyoto Encyclopedia of Genes and Genomes (KEGG) [16] enrichment analysis was performed using KOBAS [17]. We chose the hypergeometric test/Fisher’s exact test as the statistical method and QVALUE as the FDR correction method. KEGG terms with a corrected *p* value of < 0.05 were considered to be significantly enriched.

#### Motif analysis

Phosphorylated peptides were submitted to MoMo modification motifs (http://meme-suite.org/tools/momo) [18] to obtain the sequence characteristics using the motif-x algorithm.

#### Kinase prediction and kinase-substrate interaction analysis

All the identified phosphosites were uploaded to NetworKIN [19] for kinase prediction following the algorithm’s instructions. Based on the Gene Set Enrichment Analysis (GSEA) principle [20], kinase activity was calculated and matched to the human kinome tree. The kinome tree was modified courtesy of Cell Signalling Technology Inc. (www.cellsignal.com) and annotated using Kinome Render [21].

For strict kinase-substrate analysis, a network among proteins with differentially expressed phosphosites was constructed according to the manually curated PhosphoSitePlus database [22]. We used Cytoscape software to present the kinase-substrate interactions.

### Transcriptome and analysis

RNA isolation, library preparation, and sequencing were performed by Novogene Bioinformatics Technology Co., Ltd. (Tianjin, China). Briefly, a total of 3  g of RNA per sample was used as input material for RNA sample preparation. First, ribosomal RNA was removed with the Epicentre Ribo-zero™ rRNA Removal Kit (RZH1046, Epicentre, USA), and rRNA-free residue was cleaned up by ethanol precipitation. Subsequently, sequencing libraries were generated using rRNA-depleted RNA with the NEBNext® Ultra™ Directional RNA Library Prep Kit for Illumina® (NEBE7770, NEB, USA) following the manufacturer’s recommendations. Fragmentation was carried out using divalent cations under elevated temperature in NEBNext First Strand Synthesis Reaction Buffer (5 ×). First-strand cDNA was synthesized using random hexamer primers and M-MuLV Reverse Transcriptase (RNase H). Second-strand cDNA synthesis was subsequently performed using DNA polymerase I and RNase H. In the reaction buffer, dTTP was replaced by dUTP. The remaining overhangs were converted into blunt ends via exonuclease/polymerase activities. After adenylation of the 3’ ends of DNA fragments, the NEBNext Adapter with a hairpin loop structure was ligated to prepare for hybridization. To preferentially select cDNA fragments 150 ~ 200 bp in length, library fragments were purified with an AMPure XP system (Beckman Coulter, Beverly, USA). Next, 3 μL of USER Enzyme (NEB, USA) was used with size-selected, adaptor-ligated cDNA at 37 ℃ for 15 min followed by 5 min at 95 ℃ before PCR. PCR was performed with Phusion High-Fidelity DNA polymerase, Universal PCR primers, and Index (X) Primer. Finally, the products were purified (AMPure XP system) and library quality was assessed on an Agilent Bioanalyzer 2100 system. Clustering of the index-coded samples was performed on a cBot Cluster Generation System using TruSeq PE Cluster Kit v3-cBot-HS (Illumina) according to the manufacturer’s instructions. After cluster generation, the libraries were sequenced on an Illumina HiSeq 4000 instrument, and 150 base pairs and paired-end reads were generated. For quality control, raw data in fastq format were first processed using Novogene Perl scripts. Clean data were obtained by removing reads containing adapters, reads containing poly-N, and low-quality reads from the raw data. In addition, the Q20, Q30, and GC contents of the clean data were calculated. All downstream analyses were based on the high-quality clean data. RNA sequencing data were deposited in the Sequence Read Archive under the BioProject ID PRJNA609589.

Reference genome and gene model annotation files were downloaded from the Ensembl website (genome: ftp://ftp.ensembl.org/pub/release-97/fasta/mus_musculus/dna/Mus_musculus.GRCm38.dna.primary_assembly.fa.gz; gtf: ftp://ftp.ensembl.org/pub/release-97/gtf/mus_musculus/Mus_musculus.GRCm38.97.gtf.gz). HISAT2 (v2.0.5) was used to build the reference genome index and align paired-end clean reads to the reference genome. Then, StringTie (v1.3.3) was used to assemble the mapped reads of each sample and calculate the FPKM values of coding genes. FPKM means fragments per kilobase of exon per million fragments mapped, calculated based on the length of the fragments and the read count mapped to each fragment. Transcripts with FPKM values > 1 in over 50% of the samples in either group were considered validated. The edgeR R package (v3.243) provided statistical routines for determining differential expression in digital transcript or gene expression data using a model based on a negative binomial distribution. Transcripts with adjusted *p* values < 0.05 were considered to be differentially expressed. Up- and downregulated transcripts were determined based on the log2 FC (generated by edgeR, mean values of old mice/mean values of young mice) > 0 or < 0, respectively.

### Ingenuity pathway analysis (IPA) location and type analyses

IPA is a continuously updated commercial database with some distinctive analysis modules. “Location” and “Type” are the most common annotations derived from IPA. We think “Type” may represent the basic functional property of each protein. To obtain an initial general impression of the proteins identified in this study, we presented IPA location and type analyses for the proteins and transcripts identified in this study.

### Principal component analysis (PCA)

Phosphosites/transcripts quantifiable in all samples were selected for PCA. We used the “Principal component analysis” function in Perseus software and the “prcomp” function in R to perform PCA. The PCA graphics were finally generated by R.

### Transcription factor (TF)-target interaction analysis

TRRUST collects 6,552 TF-target interactions for 828 mouse TFs [23]. According to the trust_rawdata.mouse.tsv file downloaded on 2021/01/08, we selected TFs from phosphoproteome-identified proteins with differentially expressed phosphosites and targets from transcriptome-identified differentially expressed transcripts. The TF-target interaction network was constructed by Cytoscape software.

## Results

### General condition of young and old mice and workflow of phosphoproteome analysis

**Fig. 1:**
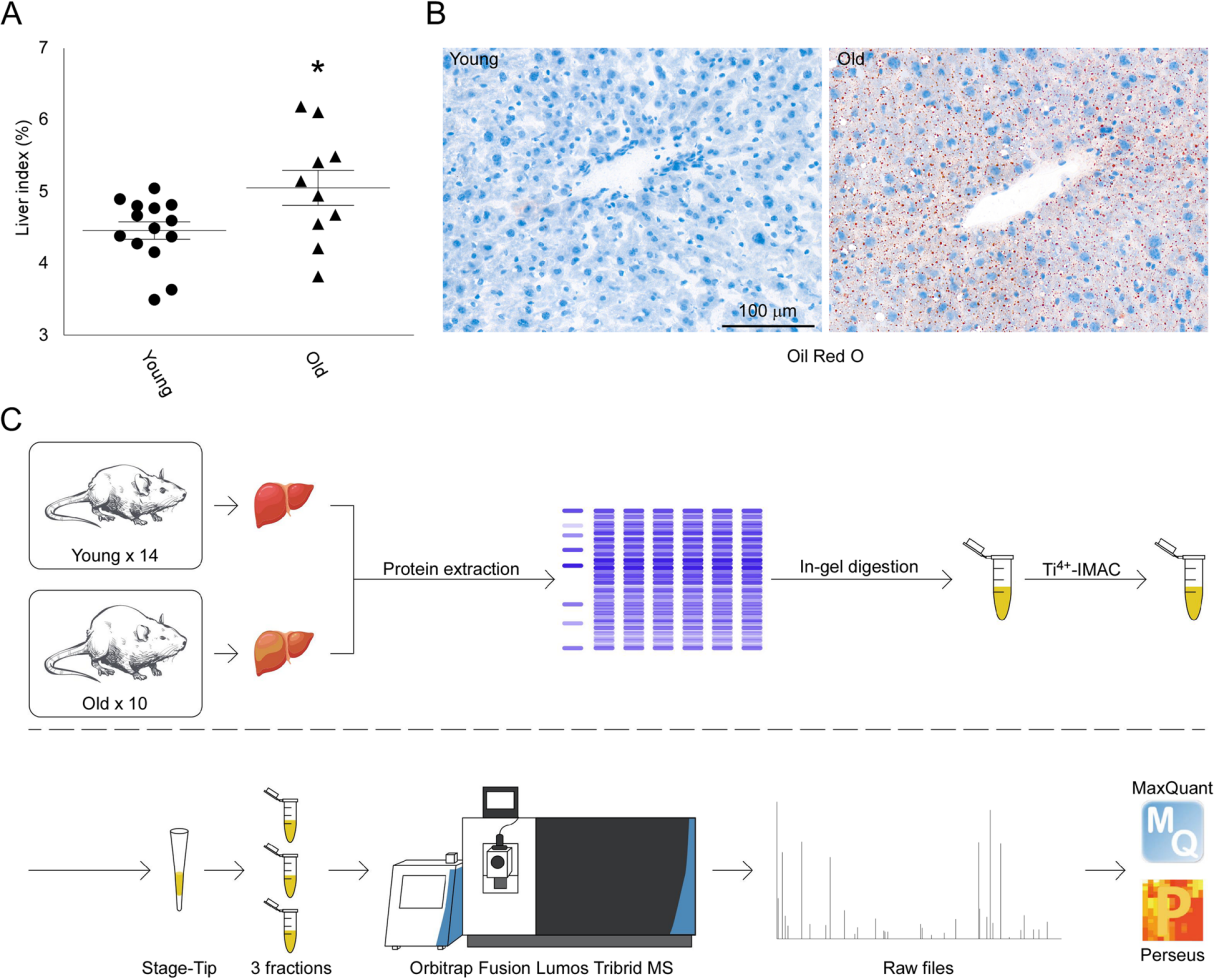
General description of mouse liver during normal aging and workflow of the phosphoproteome detection process. **A** Liver index of mice (young: *n* = 14; old: *n* = 10; mean ± standard error of the mean shown; *t test, *p* < 0.05). **B** Oil red O staining of young and old mouse livers. **C** Workflow of the phosphoproteome detection process

Fourteen 2-month-old young mice and ten 18-month-old aged mice were euthanized in this study. The aged mice weighed 32.72 ± 1.937 g, which was significantly heavier than the young mice (25.79 ± 0.482 g; Fig. S1A). The liver index (liver weight/body weight × 100%) of the old mice was 5.055 ± 0.2444%, slightly higher than that of the young mice (4.459 ± 0.1218%; Fig. 1A). Oil red O staining showed clear fat accumulation in old mouse livers but not in young mouse livers (Fig. 1B), which was consistent with a previous report [24]. However, H&E and Masson staining showed no obvious differences between the livers of young and old mice (Fig. S1B).

For phosphoproteome analysis, livers were collected from young and old mice and rapidly frozen. Case-by-case trypsin digestion and Ti^4+^-IMAC enrichment for phosphopeptides were processed using each tissue homogenate. The phosphopeptides from each mouse were then separated into 3 fractions and measured in a high-resolution quadrupole-Orbitrap MS. Raw MS files were analyzed by MaxQuant for peptide identification, label-free quantification, and phosphosite localization (Fig. 1C).

### Global phosphoproteome profiling of livers in young and old mice

We checked the identified spectra and peptides for phosphorylated proteins (Fig. S2A), peptide length (Fig. S2B), mass error (Fig. S2C), and Andromeda score distribution (Fig. S2D) for quality control. After exclusion of potential contaminants and reverse peptides, 17,424 unique phosphopeptides corresponding to 14,649 unique phosphosites were matched to 3,736 proteins (Fig. S2A). We retained 10,091 sites with localization probabilities > 0.75 for quantitative filtration (Fig. S2E). Finally, 5,685 phosphosites, quantified in at least 50% of young mice or old mice (Table S1), were used for statistical analysis.

**Fig. 2:**
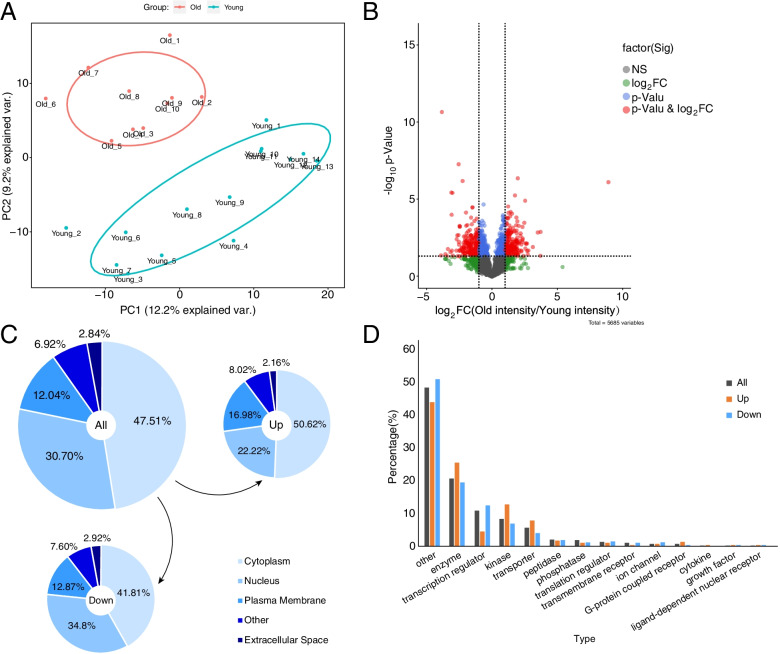
Global phosphoproteome profiling of livers in young and old mice. **A** PCA of filtered phosphosites in the livers of young and old mice. **B** FC and *P* value (two-sample *t* test) of each filtered phosphosite were plotted in a volcano plot. **C** Proteins with phosphosites were submitted to Ingenuity Pathway Analysis (IPA) for location prediction. Total: proteins corresponding to the filtered 5,685 sites. Up: proteins corresponding to the 430 upregulated sites. Down: proteins corresponding to the 493 downregulated sites. **D** Proteins with phosphosites were submitted to IPA for type prediction. Total: proteins corresponding to the filtered 5,685 sites. Up: proteins corresponding to the 430 upregulated sites. Down: proteins corresponding to the 493 downregulated sites

Principal component analysis (PCA) showed that the overall phosphorylation state of livers from old mice was to some extent distinguishable from that of livers from young mice (Fig. 2A). Using a two-sample *t* test, we identified 923 phosphosites in 633 proteins that were statistically significantly regulated during aging, including 430 upregulated sites and 493 downregulated sites (Table S2). The differences between the means of each phosphosite in old and young mice are plotted with the corresponding *P* value in Fig. 2B. We uploaded phosphorylated proteins to IPA for protein localization and type analysis. Compared to proteins with downregulated phosphosites, proteins with upregulated phosphosites were more frequently located in the cytoplasm but less frequently in the nucleus (Fig. 2C), and they contained an increased proportion of enzymes, kinases, and transporters but a decreased proportion of transcription regulators (Fig. 2D).

### Integration of altered pathways in mouse liver during normal aging

**Fig. 3:**
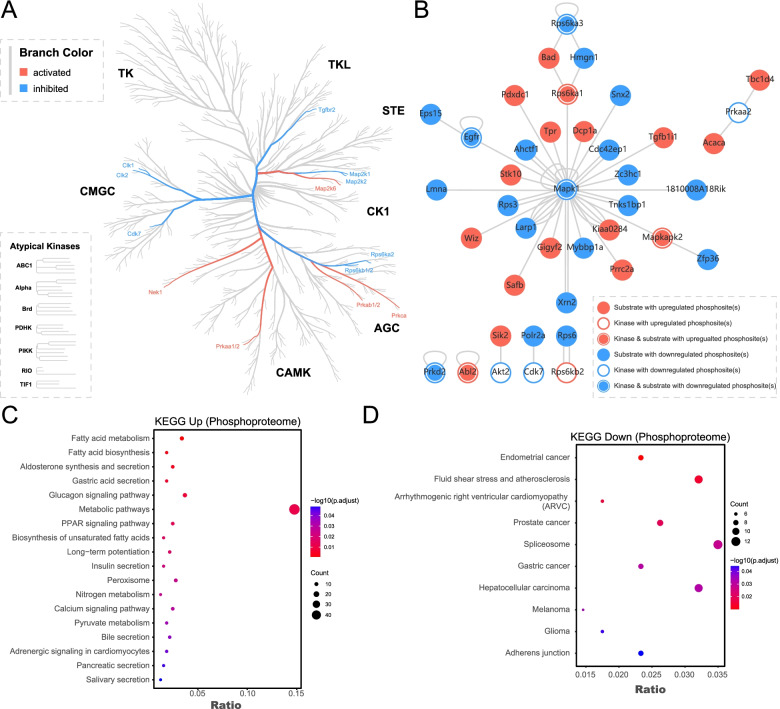
Construction of phosphorylation-associated pathway network in mouse liver at different ages. **A** Predicted kinase activity based on NetworKIN was mapped to a human kinome tree. **B** Kinase-substrate interactions among the proteins with differentially expressed phosphosites were constructed based on PhosphoSitePlus using Cytoscape software. **C** Enriched KEGG pathways for proteins with upregulated phosphosites. **D** Enriched KEGG pathways for proteins with downregulated phosphosites

**Fig. 4:**
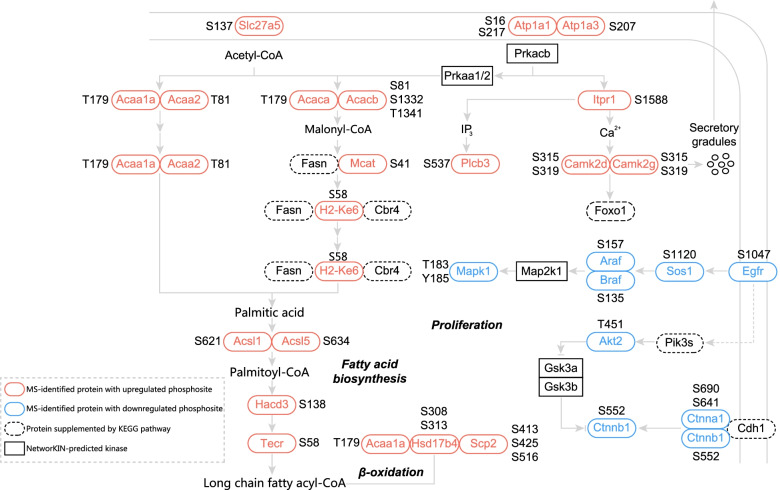
Integrated alteration of pathways in the liver of mice during normal aging. Phosphorylation-associated signaling pathways among proteins with differentially expressed phosphosites based on NetworKIN, PhosphoSitePlus, and KEGG. The identified phosphosites are annotated next to the proteins.

To comprehensively identifythe phosphorylation-regulated pathways, we performed a three-step pathway construction. First, we uploaded all the identified phosphosites to NetworKIN [19] for kinase prediction following the algorithm’s instructions. Based on the GSEA principle [20], we obtained the putative activity of each predicted kinase, finding activated Mkks/Map2k6, Prkab1/2, Prkca, Prkaa1, and Nek1 and repressed Tgfbr2, Map2k1, Map2k2, Rps6ka2, Rps6kb1, Cdk7, Clk1, and Clk2 (Fig. 3A). A previous report showed that the XpSPX motif is likely to be phosphorylated by mitogen-activated protein kinases (MAPKs), while RXXpS and RSXpS are likely phosphorylated by protein kinase C (PKC) and protein kinase A (PKA) [22, 25]. We utilized the Motif-X algorithm to obtain the characteristic motifs of up- and downregulated phosphosites but found that the relationships between predicted kinase activity and identified motifs did not exactly match those recorded in the PhosphoSitePlus database (Fig. S2F) [22]. We therefore used PhosphoSitePlus, a continuously updated database containing manually curated kinase-substrate interactions [22], to perform a second step of pathway construction. Among the proteins containing differentially regulated phosphosites, we observed a concise kinase-substrate network, including Egfr-Mapk1 and Prkaa2-Acaca (Fig. 3B). Then, for step three, we performed KEGG enrichment analysis using proteins with up- and downregulated phosphosites. We found that the aging-associated increase in phosphorylation was related to metabolism and secretion (Fig. 3C) and that aging-associated decreased phosphorylation was related to cancer, which is characterized by an abnormal cell cycle/proliferation (Fig. 3D). Finally, combined with the above three-step analysis, we constructed a phosphorylation-associated signaling network in mouse liver during aging (Fig. 4). Among the proteins with upregulated phosphosites, we found ones related to fatty acid biosynthesis, β-oxidation, and potential Ca^2+^-associated secretory processes. Among proteins with downregulated phosphosites, we identified Egfr-Sos1-Araf/Braf-Map2k1-Mapk1 and the potential Ctnnb1 pathway, both related to cell survival and proliferation [26–28].

### Potential extended transcription factor-target relationships between the phosphoproteome and transcriptome in mouse liver during normal aging

We also tried to link phosphorylation with other important regulatory elements, such as transcription factors (TFs). We performed transcriptome analysis of livers from 8 young mice and 9 old mice from the same batch of mice used for phosphoproteome analysis (Fig. S3A) and identified 13,275 transcripts (brief quality control metrics in Tables S3 and Table S4). Similar to a previous report, the transcriptome distinguished livers from young and old mice [29], which was confirmed by hierarchical clustering (Fig. S3B) and PCA (Fig. S3C). The FC and adjusted *P* value of each transcript were calculated using the edgeR R package and plotted in Fig. S3D. The differentially expressed transcripts (813 upregulated and 629 downregulated transcripts) were enriched for metabolic pathways, but fatty metabolism was not prominent (Figs. S3E, S3F). TRRUST collects 6,552 TF-target interactions for 828 mouse TFs [23]. Based on TRRUST, 10 proteins with differentially expressed phosphosites in the phosphoproteome were identified as TFs (e.g., Hnf4a and Ctnnb1), and they had 20 corresponding downstream transcripts in the transcriptome (Fig. S4). We observed that Ctnnb1 and Hnf4a were widely associated with multiple transcripts (low phosphorylation of Ctnnb1 was associated with increased transcription of Tbx3, Cyp2b10, Pdgfra, and Axin2 and decreased transcription of Foxa2, Vegfa, Grem2, and Cdh1; high phosphorylation of Hnf4a was associated with increased transcription of Apoa4 and Cyp2b9 and decreased transcription of Foxa1, Foxa2, Onecut1, Cyp4a12b, Cyp3a11, and Gstp1). Low phosphorylation of Ybx1 was related to decreased transcription of Egfr, and high phosphorylation of Gtf2i was related to increased transcription of Tgfbr2. We calculated Pearson correlation coefficients between phosphosites and transcripts (Table S5). Although complex, our results provided some clues for potential relationships between phosphorylated TFs and their downstream transcripts.

## Discussion

In this study, we performed LC–MS based phosphoproteome analysis of the livers of fourteen 2-month-old and ten 18-month-old mice. We constructed a phosphorylation-associated signaling network in mouse liver at these different ages, showing aging-associated increases in the phosphorylation of proteins involved in fatty acid biosynthesis, β-oxidation, and potential Ca^2+^-associated secretory processes, together with decreased phosphorylation of Egfr-Sos1-Araf/Braf-Map2k1-Mapk1 and the potential Ctnnb1 pathway.

A previous report revealed that cellular senescence, a permanent cell cycle arrest state accompanied by a secretory phenotype, exacerbates aging [2]. Elimination of senescent cells has presented promising therapeutic effects on aging-associated disorders [30], including hepatic steatosis [24]. In addition to previously reported increased Cdkn2a/p16(Ink4a) [31], our study suggested low phosphorylation of Egfr, Mapk1, and Ctnnb1, which are related to cell survival and proliferation [26–28], might promote senescence and might be targets for aging intervention.

In addition to the general metabolism- and inflammation-related processes identified by previous aging-associated transcriptome and proteome research [8, 32, 33], the phosphoproteome seemed to provide a distinctive profile of aging, presenting potentially dysregulated fatty acid metabolism, secretory processes, and cell cycle/proliferation. Moreover, phosphoproteome-derived kinase analysis extends our understanding of phosphorylation and aging. Previous reports have shown that some kinases can affect aging in multiple models and that intervention in the phosphorylation state or kinase activity might ameliorate aging. In *C. elegans*, p21-activated kinase 1 (pak-1) was found to limit lifespan by inhibiting daf-16, and inhibition of pak-1 might extend the lifespan and health span of *C. elegans* [34]. Depletion of PAK2/Pak2 delayed senescence in IMR90 human fibroblasts and mouse embryonic fibroblasts and extended lifespan in BubR1 progeroid mice [35]. Increased activity of the p38 Mapk-p53 pathway could induce decreases in the number and activity of intestinal stem cells, leading to gut stem cell aging [36]. Altered nutrient sensing is considered one of the hallmarks of cellular aging [2, 37]. Moreover, 5’-AMP-activated protein kinases (AMPK, Prkaa1 or Prkaa2 in mouse) are central sensors of nutrients that modulates Mtor signaling and activates Foxo transcription factors and Sirt1 [38]. Multiple lines of evidence show that metformin, an activator of AMPK signaling, attenuates the hallmarks of aging [39, 40]. Recently, it has been found that sorafenib activates AMPK and ameliorates nonalcoholic steatohepatitis, an aging-associated hepatic functional decline [41]. Jak/Stat and Rps6kb pathways were also dysregulated in aging, and some of their inhibitors have been approved by the US Food and Drug Administration [42]. The phosphoproteome provides a global view of altered phosphosites, pathways, and kinases at one time. We think this mouse liver- and phosphoproteome-based natural aging study could provide solid datasets and new prospects for further aging research.

At last, we list some issues that deserve further discussion here. First, although most phosphosites in the constructed phosphorylated network have been recorded in PhosphoSitePlus, the evidence is mostly from MS experiments. Functional experimentation is still needed for phosphosite annotation. Second, label-free proteome and transcriptome analysis from one-by-one matched samples would be helpful to understand the role of phosphorylation and the importance of phosphoproteomics. In this study, non-availability of one-by-one matched label-free whole proteome and trancriptome data for these samples is a lacuna. Moreover, label-free proteome and some post translational modification-proteome analyses, such as ubiquitylome and sumoylome may need more attention in future researches. We believe that further comprehensive multi-omics analyses with advanced technologies may provide much more abundant information for data mining. Third, 24 male mice were euthanized by tribromoethanol, but the influence of sex and anesthetics on this experimental system was difficult to assess in this study. It is important to investigate other organs and both sexes in future studies to avoid biases [43] and to obtain a comprehensive profile of liver aging and systematic aging.

Overall, our study obtained the first phosphoproteome profiling of mouse livers using LC–MS/MS during normal aging. A global view of tissues with advanced omics technology may help us identify integrated mechanisms of aging and thus find potential intervention methods.

## Conclusions

In summary, we constructed a phosphorylation-associated network in the livers of mice at different points during normal aging, which may help to discover novel antiaging strategies.

## Supplementary Information

**Additional file 1:Fig. S1.** Supplementalinformation on the general condition of young and old mice. (A) Body weight ofmice (young: *n* =14; old: *n* = 10; mean ± standard error of the mean shown;***two-sample *t* test, *p* < 0.001). (B)Morphological analysis of young and old mouse livers. Left panel: H&Estaining. Right panel: Masson staining.

**Additional file 2:Fig. S2.** LC–MS/MS quality control of the phosphoproteome. (A) Identifiedspectra and peptides of phosphorylated proteins. (B) Peptide lengthdistribution of phosphorylated peptides. (C) Mass error distribution ofphosphorylated peptides. (D) Andromeda score distribution of phosphorylatedpeptides. (E) Phosphorylated sites can be classified into four groups based onlocalization probability, which ranges from 0 to 1. The class-localizationprobability relationship is Class I-0.75~1; Class II-0.5~0.75; Class III-0.25-0.5;Class IV-0~0.25. The proportion of phosphorylated amino acids in Class I wascalculated and is presented in a pie chart. (F) Representative motifs ofdifferentially expressed phosphosites. Up: the 430 upregulated phosphosites.Down: the 493 downregulated phosphosites.

**Additional file 3:Fig. S3.** Basic analysisof the transcriptome of livers from young and old mice. We performedtranscriptome analysis of livers from 8 young mice and 9 old mice andidentified 13,275 transcripts, including 813 upregulated and 629 downregulatedones. See also Table S4. (A) Correspondence between the samples used forphosphoproteome and transcriptome analyses. (B) Hierarchical clusteringanalysis of all 13,275 transcripts in the livers of young and old mice(clustering distance = Euclidean, clustering method = complete). (C) PCA oftranscripts in the livers of young and old mice. (D) Volcano plot of the FC and*P* value (two-sample *t *test) of each transcript. (E) Top tenenriched KEGG pathways for upregulated transcripts. (F) Top ten enriched KEGGpathways for downregulated transcripts.

**Additional file 4:Fig. S4.** TF-targetinteractions based on TRRUST. TFs were chosen from proteins with differentiallyexpressed phosphosites identified in the phosphoproteome. Targets of TFs werechosen from differentially expressed transcripts identified in thetranscriptome. The TF-target network was constructed based on the TRRUSTdatabase using Cytoscape software. Hexagon: proteins with differentiallyexpressed phosphosites in the phosphoproteome (red edge: the protein containsupregulated phosphosites; blue edge: the protein contains downregulatedphosphosites). Circle: differentially expressed transcripts in thetranscriptome (pink fill: upregulated transcripts; cyan fill: downregulatedtranscripts). 

**Additional file 5:Table S1.** Quantified 5685 phosphosites

**Additional file 6:Table S2.** Differentially expressed 923 phosphosites

**Additional file 7:Table S3.** Quality control metrics for transcriptome

**Additional file 8:Table S4.** Quantified 13275 transcripts

**Additional file 9:Table S5.** TF-target interactions based on TRRUST

## Data Availability

Raw phosphoproteome data are deposited in the ProteomeXchange Consortium under the identifier PXD024270. Raw transcriptome data are deposited in the Sequence Read Archive under the ID PRJNA609589.
