# Supplementary figures and images for: Phosphoproteome profiling of mouse liver during normal aging
Source: Proteome Sci. 2022 Aug 5;20:12. doi: 10.1186/s12953-022-00194-2 (PMC9354360; doi:10.1186/s12953-022-00194-2)

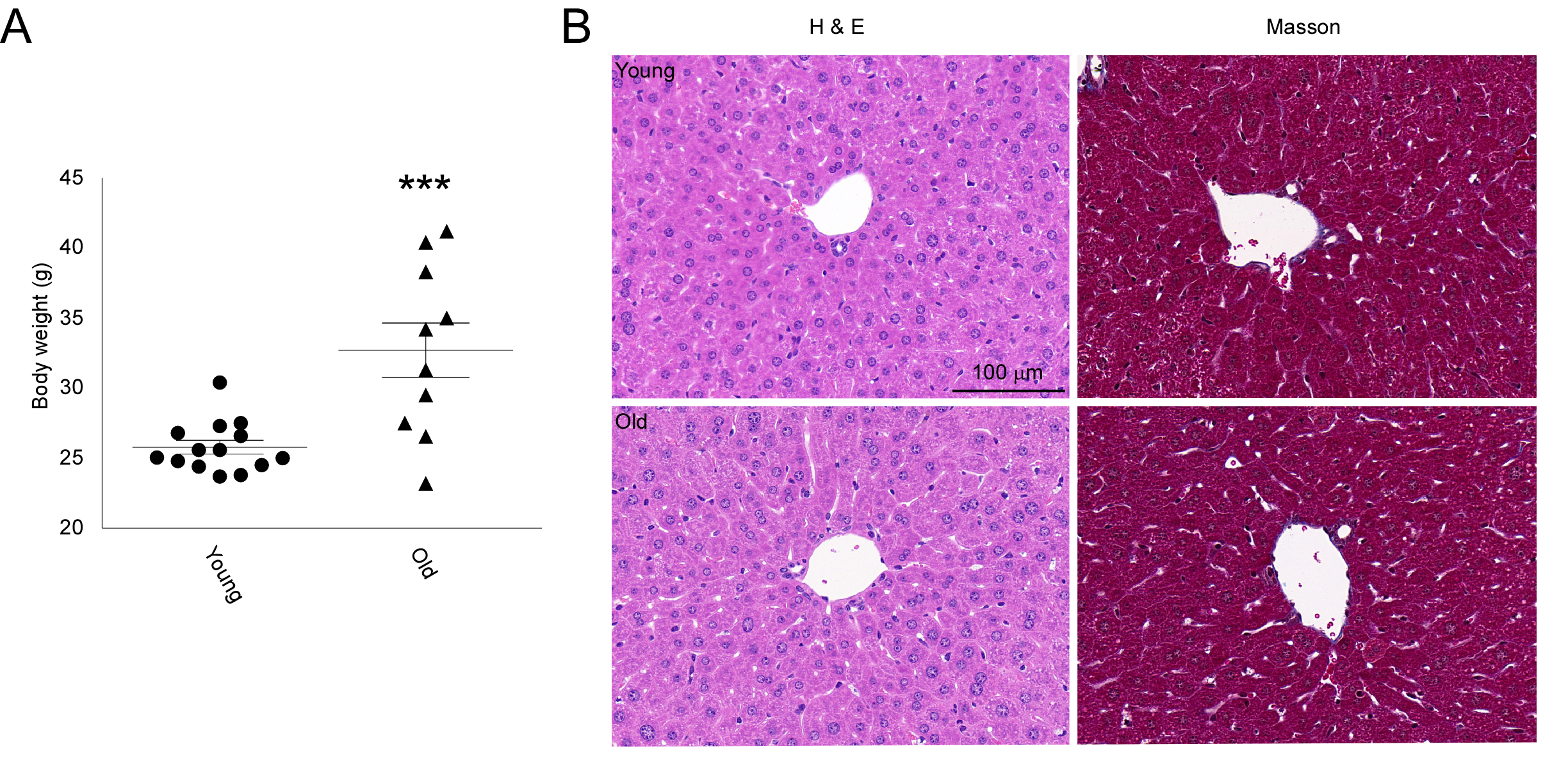

Supplement: Supplementary file 1 — Additional file 1:Fig. S1. Supplementalinformation on the general condition of young and old mice. (A) Body weight ofmice (young: n =14; old: n = 10; mean ± standard error of the mean shown;***two-sample t test, p < 0.001). (B)Morphological analysis of young and old mouse livers. Left panel: H&Estaining. Right panel: Masson staining. [file 12953_2022_194_MOESM1_ESM.tif]

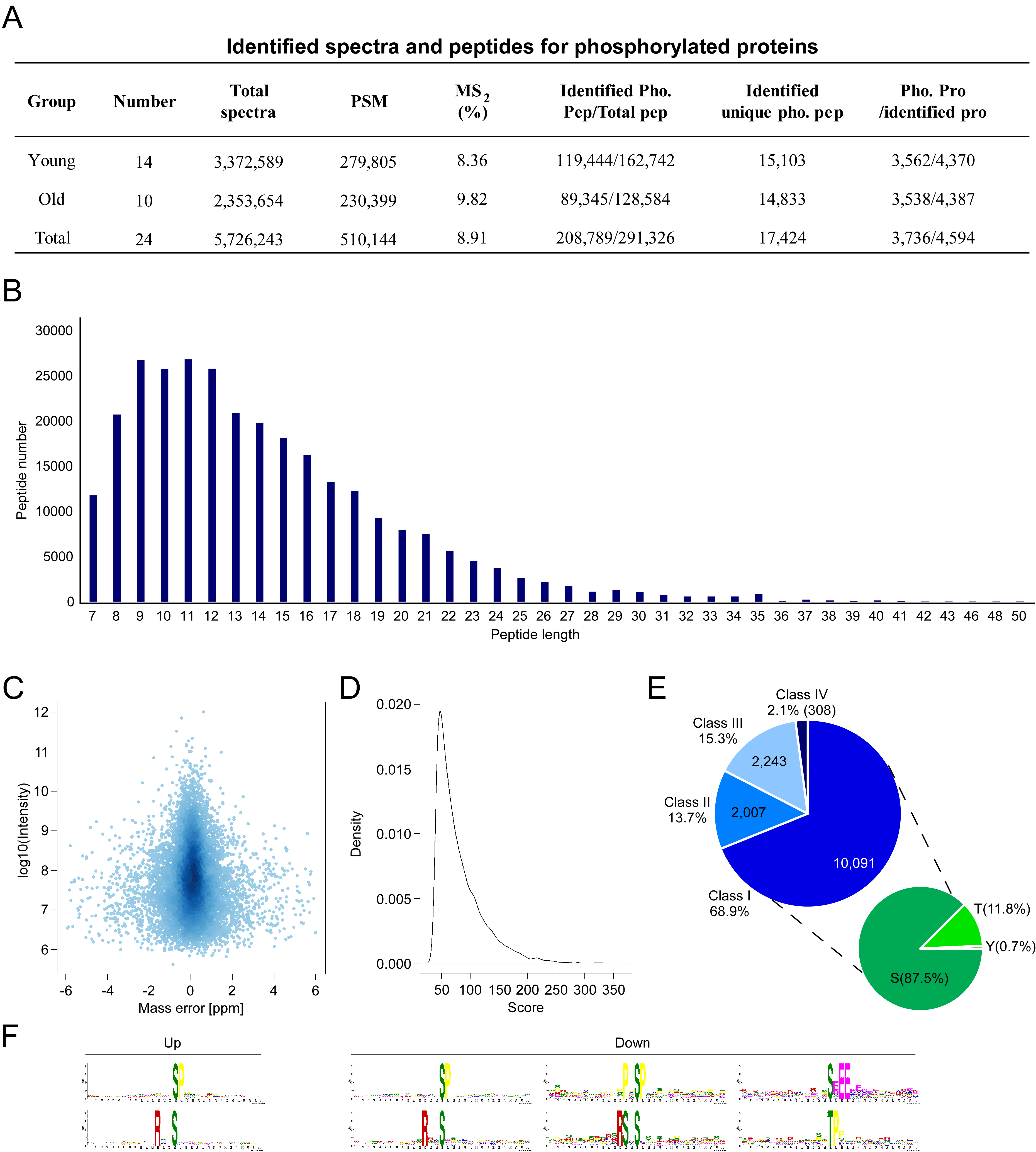

Supplement: Supplementary file 2 — Additional file 2:Fig. S2. LC–MS/MS quality control of the phosphoproteome. (A) Identifiedspectra and peptides of phosphorylated proteins. (B) Peptide lengthdistribution of phosphorylated peptides. (C) Mass error distribution ofphosphorylated peptides. (D) Andromeda score distribution of phosphorylatedpeptides. (E) Phosphorylated sites can be classified into four groups based onlocalization probability, which ranges from 0 to 1. The class-localizationprobability relationship is Class I-0.75~1; Class II-0.5~0.75; Class III-0.25-0.5;Class IV-0~0.25. The proportion of phosphorylated amino acids in Class I wascalculated and is presented in a pie chart. (F) Representative motifs ofdifferentially expressed phosphosites. Up: the 430 upregulated phosphosites.Down: the 493 downregulated phosphosites. [file 12953_2022_194_MOESM2_ESM.tif]

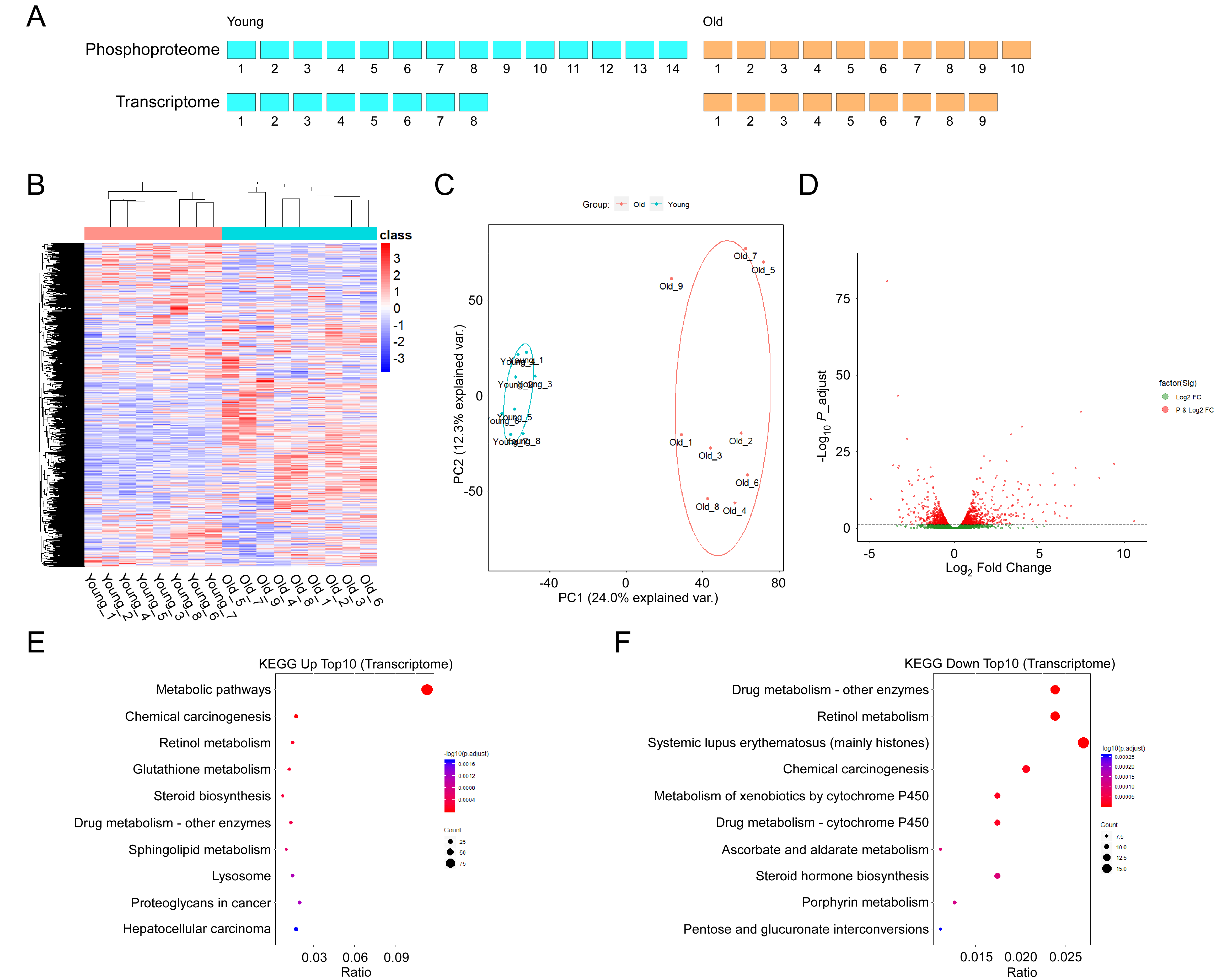

Supplement: Supplementary file 3 — Additional file 3:Fig. S3. Basic analysisof the transcriptome of livers from young and old mice. We performedtranscriptome analysis of livers from 8 young mice and 9 old mice andidentified 13,275 transcripts, including 813 upregulated and 629 downregulatedones. See also Table S4. (A) Correspondence between the samples used forphosphoproteome and transcriptome analyses. (B) Hierarchical clusteringanalysis of all 13,275 transcripts in the livers of young and old mice(clustering distance = Euclidean, clustering method = complete). (C) PCA oftranscripts in the livers of young and old mice. (D) Volcano plot of the FC andP value (two-sample t test) of each transcript. (E) Top tenenriched KEGG pathways for upregulated transcripts. (F) Top ten enriched KEGGpathways for downregulated transcripts. [file 12953_2022_194_MOESM3_ESM.tif]

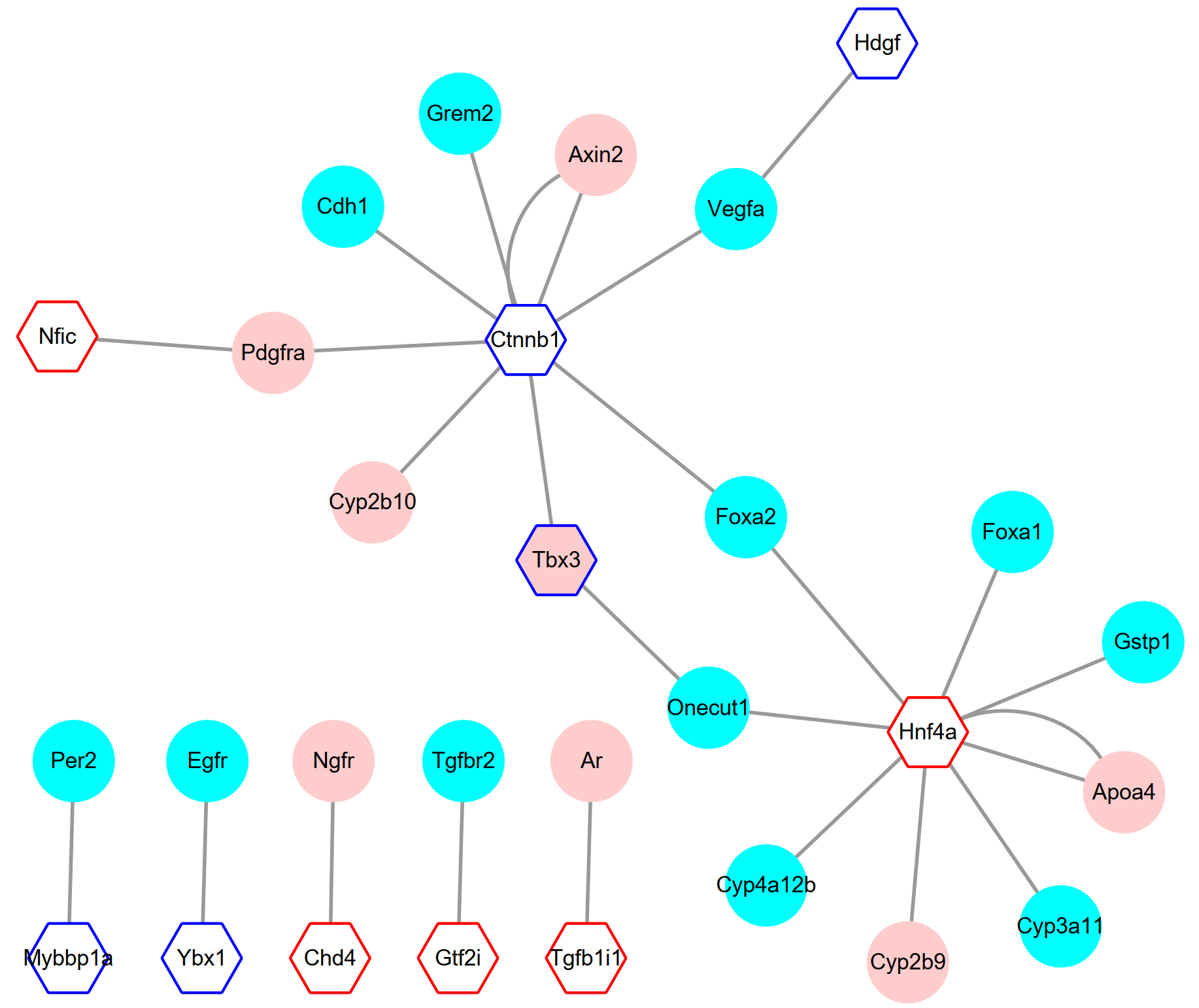

Supplement: Supplementary file 4 — Additional file 4:Fig. S4. TF-targetinteractions based on TRRUST. TFs were chosen from proteins with differentiallyexpressed phosphosites identified in the phosphoproteome. Targets of TFs werechosen from differentially expressed transcripts identified in thetranscriptome. The TF-target network was constructed based on the TRRUSTdatabase using Cytoscape software. Hexagon: proteins with differentiallyexpressed phosphosites in the phosphoproteome (red edge: the protein containsupregulated phosphosites; blue edge: the protein contains downregulatedphosphosites). Circle: differentially expressed transcripts in thetranscriptome (pink fill: upregulated transcripts; cyan fill: downregulatedtranscripts). [file 12953_2022_194_MOESM4_ESM.tif]
